# Impact of distinct neurotransmitter release modes on neuronal signaling

**DOI:** 10.1038/s41380-025-03373-7

**Published:** 2025-11-26

**Authors:** Kevin J. Zhang, Lisa M. Monteggia, Ege T. Kavalali

**Affiliations:** 1 Vanderbilt Brain Institute, Vanderbilt University, Nashville, TN USA; 2 Department of Pharmacology, Vanderbilt University, Nashville, TN USA

**Keywords:** Neuroscience, Physiology

## Abstract

Neuronal communication is governed by a diverse repertoire of neurotransmitter release modes, each with distinct molecular machinery and functional roles. Beyond rapid, high-fidelity synchronous release, asynchronous release supports sustained neurotransmitter output, while spontaneous, action-potential-independent release plays a critical role in synaptic development, homeostasis, and plasticity. Complementing these forms of release, slow neurotransmission mediated by monoamines and neuropeptides acts over longer timescales to shape network-wide activity. This review explores the unique mechanisms of each mode, highlighting compelling evidence that spontaneous and evoked release are functionally segregated through separate vesicle pools and distinct postsynaptic receptors. We also examine the molecular complexity and debated physiological roles of asynchronous release, particularly at excitatory synapses, and the specialized machinery of neuromodulatory systems. A comprehensive understanding of these varied release mechanisms is fundamental to neuroscience and opens novel therapeutic avenues. Targeting the unique molecular components of each release mode offers a promising strategy for developing more precise treatments for neurological and psychiatric disorders.

## Introduction

Neuronal communication relies on a diverse repertoire of neurotransmitter release mechanisms. While the action potential (AP) serves as the canonical trigger, the way neurotransmitters are released and the subsequent impact on target cells vary considerably across synapses and contexts. Classically, fast synaptic transmission is understood to operate via three principal modes: synchronous, asynchronous, and spontaneous release. Beyond these, the brain’s signaling landscape is shaped by slow neurotransmission, or neuromodulation, which operates on distinct principles and timescales (Table 1). This review will explore these distinct modes of release, highlighting their unique molecular machinery, regulatory mechanisms, and functional significance.

**Table 1:** Comparative summary of neurotransmitter release modes.

	Synchronous release	Asynchronous release	Spontaneous release	Neuromodulation (Volume transmission)
**Trigger**	Action potential (AP)	Residual Ca^2+^ following AP	Stochastic/no AP	AP firing pattern (tonic/phasic); spontaneous
**Key molecular machinery**	Syt-1, Syt-2, VAMP2 (also known as Syb2)	Syt-3, Syt-7, Doc2α, VAMP4	Syt-1 (acts as a clamp), Doc2β, Vti1a, VAMP7	Syt-1, Syt-7, non-canonical Syt isoforms (e.g., Syt-4, Syt-11)
**Postsynaptic target**	Ionotropic receptors	Possibly spatially distinct ionotropic receptors	Spatially distinct ionotropic receptors	GPCRs (often extrasynaptic)
**Primary cascade**	Rapid ion flux	Summated ion flux → sustained depolarization	NMDAR → eEF2K → inhibition of protein synthesis	GPCRs → G-protein → second messengers
**Key function**	High-fidelity information transfer	Sustained network activity, oscillatory activity	Homeostatic plasticity, synaptic development and maintenance	Setting network state, modulation of excitability
**Timescale**	Less than a few ms	Tens of ms to seconds	Stochastic	Seconds to minutes+

Key distinguishing characteristics of the four major modes of neurotransmitter release discussed in this review: synchronous, asynchronous, spontaneous, and neuromodulatory. Features compared include the primary trigger, key molecular machinery, principal postsynaptic targets, downstream signaling cascades, core physiological function, and characteristic timescale of action.

Synchronous release, considered the canonical form of synaptic communication, is characterized by rapid, tightly time-locked fusion of synaptic vesicles within milliseconds of an AP’s arrival at the presynaptic terminal [1–3]. This process, driven by rapid influx of calcium ions, is responsible for mediating fast, high-fidelity information transfer. Synaptotagmin-1 (Syt-1) was initially identified as a key low-affinity calcium sensor for this mode of release, working in concert with the core SNARE (soluble N-ethylmaleimide-sensitive factor attachment protein receptor) complex to drive vesicle fusion [4, 5]. However, the SNARE complex does not act in isolation; its function is critically modulated by regulatory proteins that include the Munc13 and Munc18 families, which are essential for vesicle priming [6–8], and Rab GTPases, which serve as molecular switches governing multiple stages of the synaptic vesicle cycle, from trafficking to docking [9–11]. This intricate interplay of calcium sensors, SNARE proteins, and regulatory factors ensures precise and efficient neurotransmitter release critical for synaptic function.

In contrast, asynchronous release comprises neurotransmitter release that is also AP-evoked but occurs with a significant delay that persists for tens to hundreds of milliseconds after the initial stimulus [12–15]. This mode is often enhanced during periods of high neuronal activity, contributing to a sustained barrage of neurotransmitter release. Asynchronous neurotransmission is mechanistically separable from its synchronous counterpart and often relies on different calcium sensors with higher affinity, such as Synaptotagmin-7 (Syt-7) and potentially Doc2 isoforms and distinct SNARE components [16].

**Fig. 1:**
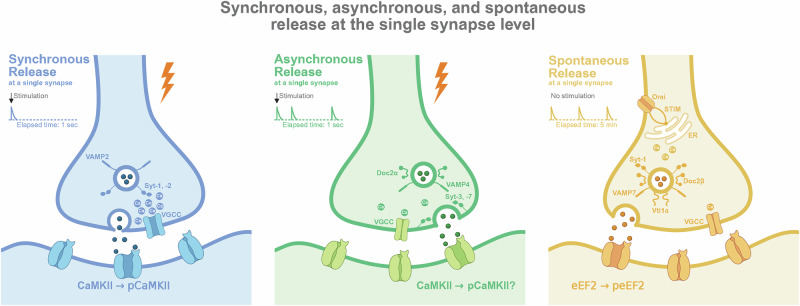
Synchronous, asynchronous, and spontaneous release at the single synapse level. Schematic representation of the three principal modes of fast synaptic transmission at a single synapse. **Presynaptically:** (Left, Blue) Synchronous release is tightly coupled to an action potential (AP). It is triggered by high, localized calcium concentrations within a nanodomain near voltage-gated calcium channels (VGCCs). The low-affinity calcium sensors Synaptotagmin-1 (Syt-1) and Synaptotagmin-2 (Syt-2) along with the core SNARE complex, drive rapid vesicle fusion. (Middle, Green) Asynchronous release is also AP-evoked but occurs with a significant delay, lasting for tens to hundreds of milliseconds or longer. It is triggered by the accumulation of lower, residual “bulk” calcium in the terminal. This mode is mediated by a distinct set of high-affinity calcium sensors, including Synaptotagmin-3 (Syt-3), Synaptotagmin-7 (Syt-7), and Doc2α, and often engages the SNARE protein VAMP4. (Right, Yellow) Spontaneous release occurs stochastically in the absence of AP stimulation. It can be triggered by local calcium signals from stochastic VGCC openings or, critically, by calcium release from internal stores such as the endoplasmic reticulum. STIM proteins sense ER calcium depletion and promote store-operated calcium entry, further supporting spontaneous release. This overall process relies on distinct molecular machinery, including Doc2β and non-canonical SNAREs like VAMP7 and Vti1a. **Postsynaptically:** Spontaneous NMDAR activation can trigger the phosphorylation of eukaryotic elongation factor 2 (eEF2), a key step in regulating local protein synthesis. The (potential) activation of the downstream kinase CaMKII is indicated for AP-driven release modes. Traces represent timing of release events.

The third classical mode, spontaneous release, occurs independently of presynaptic APs [17]. First described as “miniature end-plate potentials” at the frog neuromuscular junction, these events arise from the stochastic fusion of individual synaptic vesicles [18]. Far from being mere synaptic noise, spontaneous release plays critical roles in synaptic development, maturation, homeostasis, and plasticity [19]. The molecular machinery governing spontaneous release also shows divergence, with increasing evidence suggesting the involvement of distinct vesicle pools and alternative SNARE proteins. Of note, Syt-1, the primary calcium sensor for synchronous release can also act as a “clamp” to actively suppress spontaneous fusion at rest [20]. The core molecular and temporal features distinguishing these three classical release modes are summarized in Fig. 1.

Complementing these faster forms of synaptic transmission is the broad category of slow neurotransmission, or neuromodulatory transmission. This mode, predominantly mediated by monoamines (e.g., dopamine, serotonin), acetylcholine, and neuropeptides, operates over much longer timescales that range from seconds to minutes or even hours [21, 22]. Neuromodulators often act via G-protein-coupled receptors (GPCRs) and frequently utilize volume transmission to diffuse through the extracellular space, rather than being confined to a narrow synaptic cleft [23]. For example, dopaminergic and serotonergic systems exhibit unique release properties, including tonic and phasic firing patterns that establish ambient neurotransmitter levels or signal salient events, respectively [24–26]. Their release sites and presynaptic machinery can also differ significantly from those mediating fast neurotransmission [27].

Together, these distinct modes of neurotransmitter release (synchronous, asynchronous, spontaneous, and neuromodulatory) represent fundamental operational principles that endow neural circuits with remarkable power and adaptability. Elucidating their individual contributions and interplay is essential for understanding the complexities of brain function in both health and disease. While this review focuses on presynaptic release modes, it is crucial to acknowledge that they operate within a broader context that includes postsynaptic feedback mechanisms, such as retrograde signaling and activity-dependent receptor trafficking, as well as active regulation by perisynaptic astrocytes. The impact of these processes on neurotransmission are widely discussed in several excellent articles (e.g. [28–31]).

## Diverging function of evoked and spontaneous release

The functional segregation between evoked (synchronous and asynchronous) and spontaneous neurotransmitter release represents a paradigm shift in our understanding of synaptic communication. Once viewed as mechanistically similar processes differing only in their trigger, accumulating evidence indicates that these release modes not only serve distinct physiological roles but also engage separate postsynaptic signaling pathways.

### Molecular determinants of functionally distinct vesicle pools

A substantial body of evidence for the divergence of evoked and spontaneous release comes from research showing physically or functionally distinct pools of synaptic vesicles [32–35]. Studies have identified a resting pool of vesicles that fuse only spontaneously and do not participate in AP-driven release [32, 35]. Using fluorescent styryl dyes to label vesicle pools, Sara et al. (2005) demonstrated that vesicles loaded via spontaneous endocytosis during periods of rest (AP independent) are more likely to re-fuse spontaneously and are relatively resistant to release by AP-evoked activity. Conversely, vesicles labeled during activity-dependent recycling are preferentially released by subsequent APs [35]. This functional distinction suggests that these vesicle populations do not readily intermix or extensively “cross-talk”.

Molecular insights have come from work that identified specific proteins associated with these distinct vesicle pools. For instance, the non-canonical SNARE protein Vps10p-tail-interactor-1a (Vti1a) has been identified as a molecular marker and crucial component of a synaptic vesicle pool that recycles preferentially under resting conditions and is essential for maintaining spontaneous neurotransmission, particularly high-frequency spontaneous events [36]. Knockdown of Vti1a selectively diminished the frequency of spontaneous events without significantly affecting evoked release parameters [36]. Similarly, another non-canonical SNARE, vesicle-associated membrane protein 7 (VAMP7, also known as tetanus-insensitive VAMP or TI-VAMP), has been implicated in mediating spontaneous release from a distinct synaptic vesicle population [37–39]. In support of this premise, Crawford et al. (2017) showed that the combined loss of Vti1a and VAMP7 significantly impairs spontaneous high-frequency glutamate release but not endogenous network activity [38]. These findings build on earlier observations that VAMP7 follows a trafficking and recycling pathway distinct from other synaptic vesicle proteins [39] and further substantiate the hypothesis that spontaneous and evoked release may arise from molecularly segregated vesicle populations.

### Generation and organization of vesicle heterogeneity

The existence of functionally divergent vesicle populations raises a fundamental cell biological question: how is this heterogeneity generated and maintained within a single presynaptic terminal? A leading hypothesis is that distinct endocytic and trafficking pathways give rise to vesicles with different molecular compositions, and consequently, different functional properties [40]. For example, vesicles can be reformed through several mechanisms, including clathrin-mediated endocytosis (CME), ultrafast endocytosis, and activity-dependent bulk endocytosis (ADBE) [41]. Evidence suggests that the cargo retrieved via these pathways can differ. For instance, VAMP4, a SNARE protein structurally homologous to Synaptobrevin2 (VAMP2), is selectively retrieved via ADBE and is essential for this endocytic mode to proceed [42].

Further evidence for specialized biogenesis pathways comes from studies of adaptor protein (AP) complexes, which are critical for sorting protein cargo into transport vesicles. For example, the neuronal-specific AP-3 complex has been shown to generate a distinct subpopulation of synaptic vesicles in dopamine neurons that are tuned to respond preferentially to high-frequency stimulation [43, 44]. This demonstrates a clear molecular mechanism by which a neuron can create a functionally specialized vesicle pool. Ultimately, this molecular heterogeneity, conferred by the differential incorporation of proteins like specific SNAREs and other regulators during formation, is thought to be the primary determinant that sorts vesicles into distinct functional pools [40].

The physical organization of vesicle pools within the crowded presynaptic terminal also plays a key role in shaping their function. Traditionally, vesicle clusters, particularly the large reserve pool, were thought to be maintained by a static protein scaffold [34, 45]. However, an emerging concept proposes that these clusters may represent a distinct liquid phase within the cytoplasm that is formed through a process of liquid-liquid phase separation (LLPS) [46–48]. This liquid phase organization allows synaptic vesicle pools to remain tightly clustered yet dynamically mobile, facilitating their efficient recruitment to release sites [49]. Importantly, LLPS not only sustains synaptic vesicle pool clustering and recycling, but also contributes to the precise organization of active zone RIM1/2 and Munc13 nanoclusters [50]. LLPS thus functionally differentiates vesicle pools by maintaining distinct compartments that regulate vesicle mobility and availability [51]. Furthermore, LLPS disruption selectively impairs action potential-evoked neurotransmission while sparing spontaneous release, indicating a specific role for LLPS-mediated nanoscale organization in supporting fast, calcium-dependent evoked release [50]. These findings challenge traditional views and open new avenues for understanding the dynamic regulation of synaptic function.

The concept of distinct vesicle pools is not without debate, as some studies have reported evidence for shared vesicles or significant overlap between pools, particularly under certain experimental conditions or at specific synapse types [52–55]. The degree of segregation may also be dynamic and potentially vary with the developmental stage [56]. Nevertheless, the preponderance of evidence, particularly from studies employing molecular and optical imaging tools to track distinct vesicle populations, supports a model wherein at least a functionally specialized pool of vesicles exists to sustain spontaneous neurotransmission. This molecular distinction, conferred by proteins like Vti1a and VAMP7, likely underpins the differential trafficking, docking, and fusion properties of vesicles destined for spontaneous versus evoked exocytosis.

In addition to these non-canonical SNAREs, VAMP4, has also been shown to molecularly tag a pool of vesicles that drive asynchronous release, as well as a form of calcium-dependent spontaneous glutamate release. Biochemical studies have shown VAMP4 can form a stable complex with the SNAREs syntaxin-1 and SNAP-25 but does not interact with complexins or Syt-1, proteins essential for synchronous neurotransmission [57]. A subsequent study reported that key residues of VAMP4 that regulate its endocytosis were essential for the maintenance of VAMP4-mediated spontaneous glutamate release [58]. Moreover, high-frequency stimulation that typically triggers asynchronous release and retrieval of VAMP4 from the plasma membrane also augmented calcium-sensitive spontaneous release for up to 30 min in a VAMP4-dependent manner. Taken together, this VAMP4-mediated link between asynchronous and spontaneous excitatory neurotransmission may serve as a presynaptic substrate for synaptic plasticity, coupling distinct forms of release.

### Contrasting modes of calcium-dependent regulation

The triggering mechanisms for evoked and spontaneous release also exhibit fundamental differences in their dependence of calcium and the identity of the involved calcium sensors. Evoked synchronous release is characterized by a steep, highly cooperative relationship with calcium influx that typically requires micromolar calcium concentrations near open voltage-gated calcium channels (VGCCs) [59–61]. This rapid calcium signal is primarily transduced by low-affinity calcium sensors of the synaptotagmin family, notably Syt-1 and Syt-2 [62–67].

In contrast, spontaneous release generally displays a more linear and often lower dependence on calcium. It can be triggered by various calcium sources, including stochastic openings of VGCCs at resting membrane potential, calcium release from intracellular stores (e.g., endoplasmic reticulum), or calcium influx through tonically active channels like TRPV1 [59, 68–71]. STIM proteins facilitate store-operated calcium entry following ER depletion, further supporting spontaneous release [72]. While Syt-1 has been shown to contribute to a calcium-dependent component of spontaneous release, its role is complex. Paradoxically, genetic deletion of Syt-1 often leads to a dramatic increase in the frequency of spontaneous release [20, 64, 73]. This phenomenon suggests that Syt-1 acts as a “clamp” to suppress spontaneous fusion, and its removal unmasks an alternative, possibly more calcium-sensitive mechanism.

Building on these distinctions, proteins of the Doc2 family, particularly Doc2α and Doc2β, have emerged as strong candidates for calcium sensors that specifically mediate spontaneous release [74–77]. Groffen et al. (2010) provided initial evidence that Doc2β acts as a high-affinity calcium sensor for spontaneous release [74]. However, the precise role of Doc2 proteins remains an area of active investigation, as some studies suggest that they may also function as a lower-affinity calcium sensor and contribute more significantly to asynchronous release [16, 78]. The differential calcium sensitivities and the distinct molecular identities of the calcium sensors involved are fundamental to the functional segregation of evoked and spontaneous release pathways. For example, Syt-1’s dual capacity to activate synchronous release upon high calcium influx while simultaneously clamping spontaneous release at lower calcium levels represents a critical regulatory node [20, 64, 73, 79].

This diversity of calcium-sensing mechanisms, particularly the clamping function of Syt-1, along with likely contributions from other calcium sensors, may also contribute to the apparent dichotomy of pools. For instance, the C2 domain-containing protein copine-6, which is present in presynaptic terminals, binds to VAMP2 as well as other SNARE proteins in a calcium-dependent manner. This interaction between copine-6 and VAMP2 selectively suppresses spontaneous neurotransmission [80]. Recent work has also uncovered an additional layer of complexity, demonstrating that neuromodulators can indirectly shape spontaneous release. A study by Seibert and colleagues (2023) found that Synaptotagmin-9 (Syt-9) does not function as a direct calcium sensor for synaptic vesicle fusion but instead regulates the release of the neuropeptide substance P from dense-core vesicles in striatal neurons [81]. This release of substance P, in turn, modulates the frequency of spontaneous release events, illustrating a novel, indirect pathway through which presynaptic activity can be fine-tuned.

A recent study investigated the question of calcium regulation of distinct forms of release by imaging calcium signals within hippocampal presynaptic boutons using GCaMP8s tagged to VAMP2 [82]. This approach enabled visualization of evoked presynaptic calcium transients that derive from synchronized voltage-gated calcium channel openings, spontaneous presynaptic calcium transients that originate from ryanodine-sensitive calcium stores, and a baseline calcium signal that arises from stochastic calcium channel openings. The pharmacological manipulation of these signals, as well as photobleaching as a use-dependent method to probe their nanoscale organization, revealed that baseline calcium signals, but not spontaneous store-driven calcium signals, were the primary contributors to spontaneous glutamate release.

### Non-overlapping populations of postsynaptic receptors

**Fig. 2:**
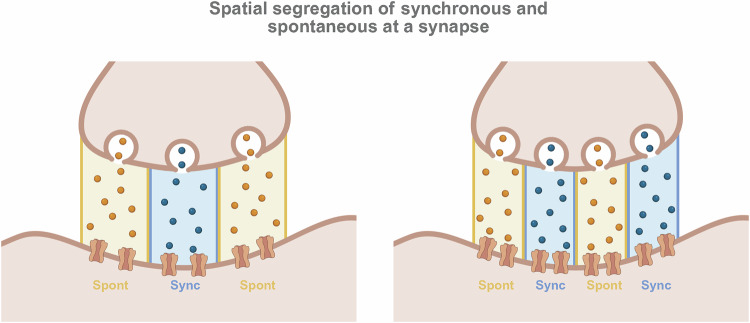
Spatial segregation of synchronous and spontaneous neurotransmitter release at a synapse. Two leading hypotheses for the spatial segregation of synchronous (Sync) and spontaneous (Spont) release through distinct nanoscale organizations at the synapse. **Left:** The center-surround model proposes a concentric arrangement where synchronous release is localized to a central domain, while spontaneous release is restricted to the surrounding periphery. **Right:** In contrast, the nanodomain model suggests that the active zone is composed of multiple, interspersed functional units, or “nanocolumns,” each specialized for either synchronous or spontaneous release through the precise trans-synaptic alignment of presynaptic release machinery and postsynaptic receptors. Both models provide a structural basis for the functional and molecular segregation of these two distinct modes of synaptic communication.

A compelling body of evidence indicates that neurotransmitters released via spontaneous versus evoked exocytosis can activate spatially segregated populations of postsynaptic receptors, even at the same synaptic site (Fig. 2). A study by Atasoy and colleagues, using the irreversible, use-dependent NMDA receptor (NMDAR) channel blocker MK-801, demonstrated that spontaneous and evoked glutamate release activate largely non-overlapping populations of NMDARs in cultured hippocampal neurons [83]. Specifically, blocking NMDARs activated by spontaneous release (by applying MK-801 at rest) had minimal impact on subsequent NMDAR-mediated evoked responses. This finding, along with subsequent studies, suggests that these two modes of release target distinct populations of NMDARs [84]. Similar segregation has been reported for AMPA receptors (AMPARs) at excitatory synapses [85–87] and, more recently, for GABA receptors at inhibitory synapses [88]. The spatial segregation of postsynaptic targets is a critical finding that provides a concrete mechanism by which spontaneous and evoked release can initiate distinct downstream signaling pathways and thereby exert independent functional effects, even when releasing the same neurotransmitter. This premise was recently bolstered by a study using photobleaching properties of iGluSnFR, a fluorescent probe that detects glutamate, to investigate the subsynaptic organization of evoked and spontaneous release in hippocampal neurons. In this setting, evoked and spontaneous iGluSnFR responses exhibited distinct photobleaching sensitivities, consistent with a subsynaptic organization in which evoked release sites are spatially clustered and associated with diffusion-restricted iGluSnFR probes, whereas spontaneous release sites are more broadly distributed and engage freely diffusible probes [89].

### Activation of unique downstream signaling pathways

Spontaneous and evoked release activate distinct receptor populations, leading to the engagement of distinct downstream intracellular signaling cascades. One of the most well-characterized examples involves the regulation of local dendritic protein synthesis. Spontaneous NMDAR activity, but notably not evoked NMDAR activity, specifically suppresses the machinery for dendritic protein translation by promoting the phosphorylation and inactivation of eukaryotic elongation factor 2 (eEF2) via eEF2 kinase (eEF2K) (Fig. 1) [90–92]. This selective signaling pathway allows spontaneous release to exert autonomous control over local protein synthesis, a process crucial for synaptic plasticity and maintenance that is independent of AP-driven activity. This capacity to engage distinct signaling pathways underscores the ability of spontaneous release to mediate unique cellular functions, rather than simply being a low-frequency echo of the evoked release machinery.

Spontaneous release also contributes to the maturation, maintenance, and stability of dendritic spines [56, 93–96]. Earlier studies showed that AMPAR activation by spontaneous vesicular glutamate release is sufficient for the maintenance of dendritic spines in hippocampal cultures [93]. This finding suggested that spontaneous events provide a crucial trophic signal for spine survival, independent of the patterned activity of synchronous release. In contrast, synchronous neurotransmission serves as the primary instructive signal for activity-dependent structural remodeling. High-frequency stimulation patterns that induce long-term potentiation (LTP) cause rapid and lasting enlargement of spine heads [97–99], whereas low-frequency stimulation leading to long-term depression (LTD) results in spine shrinkage and elimination [100–102]. Furthermore, localized, repetitive glutamate uncaging, which mimics synchronous release, can trigger the de novo formation of new, functional spines [103, 104]. While some studies suggest the initial formation of spines can occur independently of neurotransmitter release, the distinct forms of synaptic activity are vital for their long-term stability, specialization, and plastic remodeling [105, 106]. Thus, spontaneous and synchronous release together provide complementary and indispensable contributions, with each form of activity supporting the dendritic spines that shape neural circuitry.

### Spontaneous neurotransmission as a mediator of homeostatic synaptic plasticity

The continuous sensing of spontaneous neurotransmission is a key mechanism through which neurons sense overall activity levels and engage homeostatic plasticity processes that enable them to maintain stable firing rates and overall function in the face of perturbations [107–110]. One of the best-characterized forms of homeostatic plasticity is synaptic scaling, where neurons globally adjust the strength of all excitatory synapses up or down in a compensatory manner [111, 112]. This process is fundamentally activity-dependent; classic experiments using tetrodotoxin (TTX), a voltage-gated sodium channel blocker, demonstrated that chronic blockade of network activity leads to an increase in the amplitude of miniature excitatory postsynaptic currents (mEPSCs) [113, 114].

Conversely, chronically elevated activity leads to a scaling down of mEPSC amplitudes [115]. This scaling down is an active process designed to counteract hyperactivity and prevent runaway excitation. Mechanistically, it often involves the removal of postsynaptic AMPARs from the synaptic surface, which leads to a reduction of synaptic strength [114]. This process requires new gene expression and protein synthesis, engaging immediate early genes like *Homer1a* and *Arc*, which can tag synapses for endocytosis [116, 117]. Furthermore, inhibitory synapses also undergo homeostatic scaling, with hyperactivity sometimes inducing upscaling of inhibition at the soma while simultaneously downscaling inhibition at dendrites [118]. This specificity provides a sophisticated means for fine-tuning circuit stability.

Additional studies have further elucidated this process by showing that retinoic acid signaling is involved in mediating synaptic scaling induced by activity blockade, and this form of synaptic scaling is expressed postsynaptically through increased AMPAR incorporation [107, 119, 120]. Moreover, direct blockade of miniature synaptic events themselves can trigger rapid homeostatic changes, underscoring the sensitivity of neurons to this basal form of transmission [96, 109, 121].

A particularly relevant example for the field of molecular psychiatry is the mechanism of action of the rapid-acting antidepressant ketamine. Autry et al. (2011) demonstrated that by blocking NMDARs that are tonically activated by spontaneous glutamate release, ketamine leads to a de-suppression of brain-derived neurotrophic factor (BDNF) translation [90]. This occurs via the deactivation of eEF2K, which relieves its inhibitory phosphorylation of eEF2. The subsequent surge in BDNF protein promotes synaptic potentiation and is hypothesized to contribute to ketamine’s rapid antidepressant effects. Furthermore, a recent study identified a parallel signaling pathway in which spontaneous glutamate release activates metabotropic glutamate receptor 5 (mGluR5) signaling, driving rapid antidepressant effects similar to those of NMDA receptor blocker ketamine [122]. These mechanisms underscore how targeting spontaneous neurotransmission can produce a pharmacologically induced form of homeostatic plasticity and mediate behavioral changes relevant to the treatment of psychiatric conditions.

## Enigma of asynchronous neurotransmission

Asynchronous neurotransmitter release represents a fascinating and enigmatic mode of synaptic communication. While synchronous release provides the rapid and precise signaling crucial for many neural computations, asynchronous release introduces a sustained and temporally dispersed component of neurotransmitter output that significantly broadens the signaling repertoire of synapses. Although its mechanisms are distinct and its prevalence varies widely across the nervous system, the functional roles of asynchronous release are still being actively elucidated, particularly in excitatory systems.

### Molecular heterogeneity of asynchronous release

Asynchronous release is defined by its temporal characteristics: a delayed onset relative to the presynaptic AP and a prolonged duration of neurotransmitter release that can extend for tens to hundreds of milliseconds, and sometimes even seconds, after an AP. This temporal dispersion contrasts sharply with the sub-millisecond precision of synchronous release. A key feature of asynchronous release is its frequent potentiation by trains of APs or high-frequency stimulation [12, 13, 123, 124]. This activity dependence suggests that asynchronous release is often driven by the accumulation of residual calcium within the presynaptic terminal. A prominent example of this principle is found at the hippocampal mossy fiber-to-CA3 synapse, where asynchronous release can persist for seconds following high-frequency activity [125]. In addition, asynchronous release can be induced through various other methods, including elevating extracellular calcium levels, substituting extracellular calcium with strontium, and knocking down specific presynaptic proteins (e.g., Syt-1 and cysteine string protein α [CSPα]) [126–129]. However, the precise mechanistic differences underlying how these distinct conditions promote asynchronous release remain largely unexplored, highlighting a critical gap in understanding its regulation at the synaptic level.

A central hypothesis in the field is that asynchronous release is mediated by distinct presynaptic calcium sensors that possess different kinetic and affinity properties compared to the canonical synaptotagmins (Syt-1 and Syt-2) that trigger fast synchronous release. For example, Syt-7 has been extensively studied as a candidate calcium sensor for asynchronous release. Possessing an approximately ten-fold higher calcium affinity than Syt-1, Syt-7 is well-suited to respond to the lower, more persistent calcium levels that follow an AP [130]. Work by Wen et al. (2010) at the zebrafish neuromuscular junction provided strong evidence for Syt-7’s role in asynchronous release during sustained activity. Subsequent studies at various mammalian central synapses, including those by Jackman et al. (2016) and Turecek & Regehr (2018), further implicated Syt-7 in asynchronous release and related processes like synaptic facilitation [131, 132].

However, the precise contribution of Syt-7 to asynchronous release is a subject of ongoing debate. While some studies strongly support its role as a direct calcium sensor for asynchronous fusion, particularly during high-frequency stimulation, other research suggests a more indirect or supportive role. For instance, Wu et al. (2024) proposed that at excitatory mouse hippocampal synapses, Syt-7 primarily facilitates asynchronous release by promoting synaptic vesicle docking and capture after an AP, while Doc2α is specifically responsible for driving the asynchronous component of release as a presynaptic calcium sensor [16]. In their model, genetic knockout of Doc2α significantly reduced asynchronous release after a single AP, while genetic deletion of Syt-7 had no discernible effect. Adding to this complexity, another published study has suggested Synaptotagmin-3 (Syt-3) as an additional calcium sensor driving asynchronous release. The authors found that at both cerebellar and hippocampal synapses, deletion of both Syt-3 and Syt-7 led to a more significant reduction in asynchronous release compared to the deletion of either Syt-3 or Syt-7 alone [133]. This molecular complexity and the context-dependent roles of different sensors suggest that asynchronous release is not a single, uniform process but rather likely comprises a collection of release events with varying molecular underpinnings tailored to specific synaptic needs and activity patterns.

### Nanoscale organization of asynchronous fusion sites

**Fig. 3:**
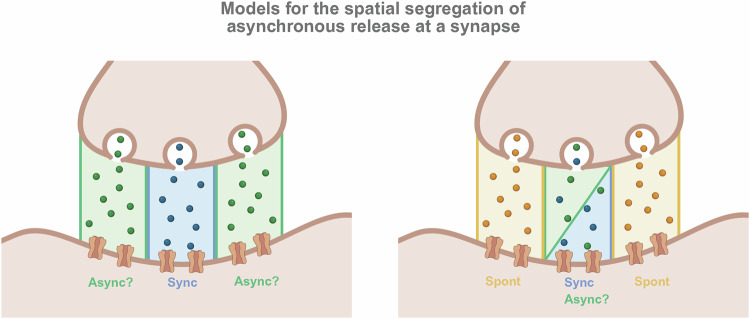
Models for the spatial organization of asynchronous neurotransmitter release at a synapse. While the segregation of synchronous and spontaneous release is increasingly understood, the precise location of asynchronous release within the active zone remains highly debated. **Left:** One hypothesis proposes a center-surround organization among evoked release modes, where fast, synchronous release (Sync) is confined to central release sites, while the more delayed asynchronous release (Async) occurs in the surrounding periphery. **Right:** An alternative model suggests that both synchronous and asynchronous release are co-localized within central release sites, while spontaneous release (Spont) is segregated to the periphery. This model is supported by findings that asynchronous and synchronous release activate the same NMDA receptors [83], implying a shared location within the active zone center, a conclusion consistent with reports of asynchronous events being biased toward this central region.

The precise location where asynchronous release occurs relative to the presynaptic active zone (AZ) and synchronous release sites remains an active and frequently debated area of research (Fig. 3). Earlier electrophysiological experiments testing the use-dependent block of distinct forms of NMDA receptor-mediated neurotransmission suggested that NMDARs activated by asynchronous and synchronous release overlap [83]. However, some studies utilizing flash-and-freeze electron microscopy have observed that asynchronous events occur preferentially closer to the center of the AZ compared to synchronous release events and are aligned with NMDARs clustered near the postsynaptic density [134, 135]. In contrast, research leveraging live optical imaging reached the opposite conclusion, finding that asynchronous events are more broadly distributed across the presynaptic area than synchronous events and cover a larger area of the AZ [136]. To help reconcile these seemingly incompatible results, another group proposed the existence of two spatially distinct subpopulations of asynchronous events: one biased towards the AZ center and another occurring ectopically outside of the functionally defined AZ [137].

The discrepancy in findings regarding the spatial organization of asynchronous release raises the important question of whether asynchronous release is a single, homogeneous phenomenon or whether it encompasses several spatially and/or temporally distinct release processes. Of note, these studies defining the spatial organization of asynchronous release employ diverse experimental approaches and assume that the mechanisms governing asynchronous release remain consistent across methodologies. Specifically, while existing literature has observed asynchronous release under various settings (e.g., native state, strontium-evoked, Syt-1 knockdown-evoked, Syt-7 mediated) [16, 134, 136, 137], to date no study has systematically explored how these manipulations may differentially influence the spatiotemporal dynamics of asynchronous release. This gap in knowledge presents a critical limitation, as the molecular and biophysical properties of asynchronous neurotransmission could be context-dependent, potentially altering synaptic output, plasticity, and information processing in ways that are not yet fully appreciated.

### Functional significance of asynchronous release at inhibitory synapses

Asynchronous release is a prominent and physiologically significant mode of release at many inhibitory synapses throughout the central nervous system. A key finding by Jonas and colleagues was the demonstration of highly asynchronous GABA release from cholecystokinin (CCK)-positive basket cells in the hippocampus, which contrasted sharply with the predominantly synchronous release from parvalbumin (PV)-positive basket cells [138]. This stark difference was attributed to distinct presynaptic calcium channel subtypes (N-type for CCK-positive neurons vs. P/Q type for PV-positive neurons), calcium buffering capacities, and the coupling between calcium sources and sensors. Importantly, the propensity for asynchronous release appears to be an intrinsic property of the presynaptic neuron, independent of its postsynaptic partner. Studies have shown that the degree of asynchrony from CCK-positive interneurons is consistent regardless of whether the target cell is a principal neuron or another interneuron [139]. This target-cell independence suggests that asynchronous release is a hard-wired feature of certain presynaptic terminals, designed to broadcast a specific type of signal throughout its local circuit. However, recent findings suggest that the transition between synchronous and asynchronous release may be plastic, with changes in excitation/inhibition balance potentially modulating release kinetics and information coding, particularly in the context of aging and neurodegenerative disorders [126].

Beyond shaping phasic inhibition, asynchronous GABA release drives tonic inhibition by spilling over from synapses to activate high-affinity, extrasynaptic GABA_A_ receptors, thereby providing a persistent, diffuse brake on network excitability [140, 141]. This process is critically regulated by the synapsin family of synaptic vesicle proteins. Genetic deletion of Synapsin II (Syn II) in mice results in a dramatic reduction of asynchronous GABA release, and leads to a near-complete loss of tonic inhibition [140, 142]. The role of Syn II is remarkably specific to the interneuron subtype; at CCK-positive interneurons, Syn II deletion selectively suppresses the asynchronous release component, whereas at PV-positive interneurons, its absence leads to a desynchronization of release [143]. This highlights the crucial role of proteins like synapsins as molecular regulators that define the balance between synchronous and asynchronous release at inhibitory synapses, thus controlling network excitability.

Work elucidating the functional significance of asynchronous release in inhibitory systems has focused on the auditory brainstem, where temporal precision is paramount. Lu and Trussell (2000) demonstrated that at GABAergic synapses in the avian cochlear nucleus (nucleus magnocellularis), neurotransmission shifts from a highly synchronized mode at physiologically low stimulus frequencies to a largely continuous and desynchronized (asynchronous) mode at physiologically high stimulus frequencies [144]. This desynchronization was shown to depend on the accumulation of intraterminal calcium and the facilitation of vesicle release. These desynchronized release events are thought to generate a smooth, sustained inhibitory “tone” that minimizes the consequences of randomly timed presynaptic APs and thereby ensuring consistent postsynaptic inhibition during intense activity.

Another compelling example of the physiological relevance of inhibitory asynchronous release is in the synapses between deep cerebellar nuclei (DCN) neurons and inferior olive (IO) neurons. Despite the DCN-IO projection playing a crucial role in cerebellar motor learning [145–147], its signaling is characterized by a lack of prominent phasic synchronous activity. Instead, these inhibitory synapses are unusually dominated by asynchronous activity across all physiologically tested frequencies [148]. This sustained asynchronous neurotransmission from DCN to IO neurons appears to support a specialized role in providing robust suppression and fine-tuned regulation of IO neuron firing and, consequently, downstream cerebellar circuits.

### The elusive physiological status of excitatory asynchronous release

While asynchronous release is a well-established and functionally significant feature of specific inhibitory synapses, its role and prevalence at excitatory synapses remain more elusive and subject to considerable debate. Much of the evidence for robust excitatory asynchronous release comes from studies involving genetic manipulations that disrupt synchronous release or from pathological conditions, rather than observations under normal physiological states.

An extensive body of research has shown that manipulations targeting Syt-1, the primary calcium sensor for synchronous release, can unmask or dramatically enhance asynchronous release at excitatory synapses. Genetic knockout or knockdown of Syt-1 not only abolishes fast synchronous release, but often leads to an increase in the slower, asynchronous component [20, 128, 149–152]. This observation has led to the model that Syt-1 not only triggers synchronous fusion but also actively suppresses or “clamps” asynchronous (and spontaneous) release pathways, which then become disinhibited in its absence. While these genetic manipulations are artificial, they reveal an intrinsic capacity for asynchronous release at excitatory terminals.

Similarly, disruption of CSPα, a synaptic-vesicle-associated co-chaperone essential for proper SNARE maintenance [153], can profoundly alter synaptic transmission. Recent work by Uzay et al. (2023) using human embryonic stem cell-derived glutamatergic neurons demonstrated that in a purely excitatory network, the emergence of NMDAR-mediated transmission can elicit endoplasmic reticulum stress [126]. This ER stress subsequently downregulates key presynaptic proteins, including Syt-1 and CSPα, leading to a progressive desynchronization of neurotransmitter release. These findings compellingly link the emergence of excitatory asynchronous release to conditions of cellular stress or excitation-inhibition imbalance and suggest that neurons may have the ability to regulate their activity and signaling in a homeostatic-dependent manner.

Furthermore, enhanced asynchronous release at excitatory synapses has been observed in animal models of certain neurodegenerative disorders, such as spinal muscular atrophy and Alzheimer’s disease [154, 155]. This shift from synchronous to asynchronous neurotransmission further hints at the possibility that prominent asynchronous release might often be a feature of synapses under duress, whether due to genetic perturbation of key presynaptic release proteins, chronic stress, or pathological conditions.

Despite its frequent association with artificial or pathological states, several potential physiological roles for excitatory asynchronous release have been proposed. One such function is the generation of sustained postsynaptic depolarization and firing [15, 156]. The temporally dispersed release of glutamate during asynchronous release can lead to the summation of excitatory postsynaptic potentials (EPSPs), causing prolonged depolarization that might be sufficient to trigger postsynaptic APs long after the initial presynaptic stimulus has ceased. This sustained excitation could be particularly effective in overcoming the post-spike afterhyperpolarization of a postsynaptic neuron, thereby shortening its relative refractory period. Beyond sustaining excitability, asynchronous release can also directly influence information transfer and the precision of neural coding. By extending the time window during which neurotransmitters are present in the cleft, asynchronous release can degrade the temporal fidelity of single spike neurotransmission. However, this same property can enhance the reliability of information transfer during high-frequency firing, effectively switching the coding strategy from one based on precise timing to one based on firing rate [44]. This suggests that the balance between synchronous and asynchronous release can dynamically tune how information is processed and relayed.

An intriguing possibility is the role of excitatory asynchronous release in the generation and maintenance of persistent network states and reverberatory activity. While long-term memory representations are thought to be stored in the synaptic connections of neuronal circuits [157, 158], short-term or working memory representations are believed to be maintained through persistent neuronal activity [159–161], often sustained by active reverberation within recurrent networks [162]. Experimental evidence in cultured hippocampal networks indicates that asynchronous release is critical for this process. Lau and Bi (2005) found that brief stimulation could evoke reverberations lasting seconds and that these reverberations were dependent on recurrent excitation mediated by AMPARs [163]. Crucially, paired-pulse stimuli that enhanced asynchronous release were more effective at triggering reverberation, while pharmacological suppression of asynchronous release with EGTA-AM abolished it. Conversely, elevating asynchronous release with strontium potentiated reverberation.

Expanding on this premise, knockdown of Doc2α, a proposed calcium sensor for asynchronous release, was found to inhibit the occurrence of persistent reverberatory activity [78]. This finding aligns with computational modeling studies, which suggest that enhanced asynchronous release, driven by residual presynaptic calcium, is a key factor for sustaining network reverberations [164]. Moreover, asynchronous release has been shown to shape network activity by, paradoxically, synchronizing neuronal ensembles under certain conditions, highlighting its complex role in modulating network dynamics [165]. The involvement of excitatory asynchronous release in reverberatory activity is a compelling candidate for a significant physiological function and suggests that, at least in some circuits, asynchronous release can contribute to emergent network properties fundamental to cognitive functions such as working memory and motor planning.

In conclusion, the study of asynchronous release is a dynamic field characterized by significant progress yet also marked by several open questions and ongoing controversies. The precise molecular identity and interplay of calcium sensors driving asynchronous release, the definitive spatial organization of asynchronous events relative to other modes of release, and the full spectrum of its physiological roles, especially at excitatory synapses under normal conditions, remain key areas for future investigation. Furthermore, understanding how different forms of asynchronous release (e.g., evoked by strontium versus residual calcium) are mechanistically related will be vital for a complete picture of this enigmatic release mode. Resolving these issues will not only deepen our fundamental understanding of synaptic transmission but may also reveal novel therapeutic targets for neurological and psychiatric disorders characterized by aberrant synaptic communication.

## Slow neurotransmission and neuromodulation

Beyond the rapid, point-to-point communication mediated by the synchronous, asynchronous, and spontaneous release of fast-acting neurotransmitters like glutamate and GABA, the nervous system employs a distinct and equally vital mode of signaling: slow neurotransmission, often termed neuromodulation. This form of communication, primarily mediated by monoamines such as dopamine and serotonin, as well as acetylcholine and a vast array of neuropeptides, operates on different principles and timescales to profoundly shape neuronal excitability, synaptic efficacy and network states.

### Distinguishing features of slow neurotransmission

Slow neurotransmission is characterized by its delayed onset and prolonged duration of action that often lasts seconds, minutes, or even longer to influence neuronal function over extended periods [166–168]. This is in sharp contrast to the rapid, millisecond-scale signaling of fast ionotropic synaptic transmission. Neuromodulation is primarily mediated by G-protein-coupled receptors (GPCRs), which, upon ligand binding, initiate intracellular second messenger cascades that influence a wide range of downstream processes, including receptor trafficking, protein synthesis, and gene expression [169–171].

A key concept associated with neuromodulation is volume transmission. Unlike classical synaptic transmission where neurotransmitters are released into a confined synaptic cleft to act on precisely apposed postsynaptic receptors, neuromodulators are often released from axonal varicosities that may lack specialized synaptic contacts [172–175]. These transmitters can then diffuse over considerable distances (micrometers to millimeters) through the extracellular space to activate receptors on multiple target cells [176–180]. This “wireless” mode of communication allows neuromodulators to exert widespread influence over entire brain regions or neuronal ensembles to coordinate their activity. Therefore, unlike fast neurotransmitters, neuromodulators do not typically convey precise, moment-to-moment information [178]. Rather, they bias, gate, or tune the processing of other inputs and the overall responsiveness of neural networks.

### Regulation and functional consequences of dopaminergic transmission

Dopamine is a quintessential neuromodulator that plays a critical role in motor control, motivation, reward processing, learning, and cognitive functions [181–185]. Synthesized mainly by neurons in the substantia nigra and ventral tegmental area, dopaminergic projections are found throughout the brain, including in the amygdala, striatum, and hippocampus [186–188]. In addition to axonal release, dopamine can also be released from the cell bodies and dendrites of dopaminergic neurons, a process referred to as somatodendritic release [189, 190].

Striatal dopamine release is often described in terms of two distinct, action potential-driven modes: tonic and phasic. Tonic release is characterized by the low-frequency firing (0.2-10 Hz) of dopaminergic neurons [191]. This mode of activity is thought to establish ambient and low concentrations of extracellular dopamine in target regions [192]. In contrast, phasic release is driven by high-frequency, synchronized firing (>10 Hz), typically elicited by salient environmental stimuli, unexpected rewards, or reward-predicting cues [193, 194]. In addition to these activity-dependent modes, dopamine can also be released spontaneously, independent of action potentials, though its physiological role remains less well understood. These distinct signaling modes arise from the release of discrete dopamine quanta that correspond to the contents of individual synaptic vesicles. The fundamental properties of these quantal events at central dopamine synapses were first directly measured using amperometry, revealing rapid, low-molecular-number release events that differ from those observed at other synapses [195]. Packing of dopamine into these vesicular quanta is mediated by the vesicular monoamine transporter 2 (VMAT2) [196, 197].

The five types of dopamine receptors (D_1_-D_5_) are grouped into two main GPCR families: D_1_-like receptors (D_1_, D_5_), which typically couple to Gα_s/olf_ G-proteins, and D_2_-like receptors (D_2_, D_3_, D_4_), which usually couple to Gα_i/o_ G-proteins [198]. Importantly, many of these receptors are located extrasynaptically, where they can be activated by diffuse dopamine [199, 200]. This spatial distribution suggests that the distinct temporal and spatial patterns of tonic and phasic release may engage different receptor populations to produce diverse downstream effects. Indeed, a very recent study has provided direct evidence for this hypothesis, revealing an unexpected level of spatiotemporal precision in striatal dopamine signaling that challenges the classical view of purely slow, volume-based transmission [201]. Using advanced optical methods, Yee and colleagues demonstrated that dopamine can be released in rapid ( ~ 100 ms), highly localized (<5 μm) “hotspots.” These high-concentration hotspots engage a fast, membrane-delimited Gβγ pathway for precise, “synaptic-like” modulation, while lower, ambient dopamine levels activate a slower, diffuse cAMP pathway to set a broader network tone [201]. This enables a dual-mode signaling system through the D_2_ receptor, where two parallel pathways operate on different spatiotemporal scales.

Together, these findings highlight the importance of understanding how dopamine release is regulated at the presynaptic side. While dopamine release is triggered by calcium influx through VGCCs, studies have found that, unlike canonical fast synapses, which predominantly utilize Ca_v_2 channels [202, 203], dopamine release is also regulated by Ca_v_1 (L-type) and Ca_v_3 (T-type) channels [204, 205]. These differences point to the possibility of a more flexible and heterogeneous release machinery, raising questions about how calcium signals are decoded in dopamine terminals.

This divergence extends to the presynaptic calcium sensors themselves. Although the canonical calcium-binding sensors Syt-1 and Syt-7 influence dopamine release [206, 207], other studies have implicated non-canonical isoforms that lack calcium-binding ability, such as Synaptotagmin-4 (Syt-4) and Synaptotagmin-11 (Syt-11) [207–209]. The involvement of these atypical synaptotagmins suggests that dopaminergic neurons may rely on alternative molecular pathways to regulate exocytosis. These molecular differences raise the question of whether the structural organization of dopamine release sites is similarly specialized.

Recent structural and functional analyses have clarified how dopamine axons organize the molecular machinery required for evoked release. Work by Kaeser and colleagues revealed that midbrain dopaminergic neurons contain active zone-like release sites that are mechanistically distinct from those at fast synapses. Using super-resolution microscopy, Liu et al. (2018) found that, similar to fast synapses, the active zone of dopaminergic boutons contain the scaffolding proteins RIM and ELKS. However, unlike fast synapses, where deletion of RIM or ELKS reduces transmitter release between 40–80% [210–213], dopamine release is critically dependent on RIM, whereas ELKS knockout has no significant effect [214]. Surprisingly, although evoked dopamine release requires these specialized sites, only about 30% of dopamine clusters are associated with them [214]. The majority of dopamine varicosities may lack this classical architecture and instead rely on alternative mechanisms of transmitter release. Consistent with this, functional imaging using fluorescent false neurotransmitters revealed that most dopamine vesicle clusters in the striatum are functionally “silent” and fail to release their contents despite normal calcium influx [215].

### Pathway-specific regulation of neural circuits by serotonergic transmission

Serotonin is another neuromodulator involved in a vast array of physiological and psychological processes, including mood regulation, anxiety, sleep-wake cycles, and appetite [216]. Though the vast majority of serotonergic neurons reside within the raphe nuclei, their axons project diffusely throughout the brain and spinal cord [217–221]. Like dopamine, serotonin is released from axonal varicosities and primarily acts via volume transmission [222, 223]. However, recent evidence also points to more localized signaling. For instance, Sheu et al. (2022) identified specialized axo-ciliary synapses between serotonergic axons and the primary cilia of hippocampal CA1 pyramidal neurons. These synapses enable highly localized serotonin signaling that can influence nuclear processes, such as chromatin accessibility, suggesting a more targeted mode of serotonin action alongside its diffuse modulatory role [224].

Serotonergic neurons, similar to dopaminergic neurons, can exhibit both tonic (pacemaker-like) and phasic (burst) firing patterns [225, 226]. Tonic firing is associated with maintaining baseline extracellular serotonin levels that influence the overall excitability of target circuits, whereas phasic firing is triggered by specific behavioral or environmental stimuli to more selectively influence synaptic and network activity [227–229]. Studies have demonstrated that the firing mode affects the transmission mechanism; while tonic firing is associated with volume transmission, phasic firing can lead to more localized, synaptic-like transmission [230]. This duality allows the serotonergic system to exert both widespread and precise effects on neural circuits.

Serotonin exerts diverse effects by acting on an exceptionally large and complex family of receptors. To date, at least 14 distinct receptor subtypes have been identified, which are grouped into seven families (5HT_1_ through 5-HT_7_) [231]. This remarkable receptor diversity provides the molecular basis for serotonin’s ability to modulate neuronal excitability and plasticity through multiple mechanisms. It can act directly on postsynaptic serotonin receptors to alter ion channel conductance [232] or indirectly by influencing the presynaptic release of other neurotransmitters like glutamate and GABA [233, 234]. For example, presynaptic 5-HT_1B_ receptors often function as inhibitory autoreceptors or heteroreceptors that reduce transmitter release [235]. Conversely, activation of other receptor subtypes, such as presynaptic 5-HT_2A_ receptors, can enhance excitatory glutamate release [236]. This potent modulation extends to synaptic plasticity, where serotonin can influence the induction and magnitude of long-term potentiation (LTP) and long-term depression (LTD) in a receptor subtype- and circuit-specific manner [237–240]. For example, 5-HT_2A_ receptor activation in the prefrontal cortex induces LTD at excitatory synapses [241], while in the hippocampus, serotonin signaling can modulate the threshold for LTP induction [242]. Collectively, these findings highlight the multifaceted role of serotonin in influencing synaptic plasticity and memory processes.

Serotonergic modulation can also play a role in the selective tuning of synaptic inputs by refining neural computations in a context-dependent manner. In the dorsal cochlear nucleus (DCN), a brainstem region that integrates auditory and multisensory inputs, Tang and Trussell (2017) demonstrated that serotonin can differentially modulate converging inputs to the same neuron. Specifically, serotonin enhanced transmission from multisensory inputs while concurrently decreasing signaling from auditory fibers [243]. Such pathway-specific modulation by serotonin highlights its capacity to fine-tune neural circuits with a high degree of spatial and functional specificity.

## Translational relevance and pathophysiological significance

Given the fundamental role of the presynaptic release machinery in coordinating all forms of neuronal communication, it is not surprising that its dysregulation is a central feature in a wide range of debilitating brain disorders. Genetic mutations or pathological alterations in the core components that govern vesicle trafficking, docking, priming, and fusion can disrupt the delicate balance between excitation and inhibition and lead to aberrant network activity. These conditions, which stem from defects at the synapse, are increasingly referred to as “synaptopathies,” and understanding their presynaptic origins is critical for developing targeted therapies.

### Epilepsy and “SNAREopathies”

Epilepsy, characterized by recurrent seizures arising from network hyperexcitability, has strong ties to presynaptic dysfunction. A growing class of severe, early-onset developmental and epileptic encephalopathies has been linked to de novo mutations in genes encoding the core components of the release machinery and has led to a group of disorders now termed “SNAREopathies” [244]. Pathogenetic variants have been identified in genes for all three neuronal SNAREs as well as their critical regulators, such as Munc18-1 [245–247]. These mutations can profoundly alter neurotransmitter release dynamics. For instance, studies of epilepsy-associated SNAP25 mutations have revealed that they can specifically augment spontaneous release, suggesting that an imbalance between distinct release modes, rather than a simple loss of function, can be sufficient to drive a pathogenic, hyperexcitable state [248, 249].

**Fig. 4:**
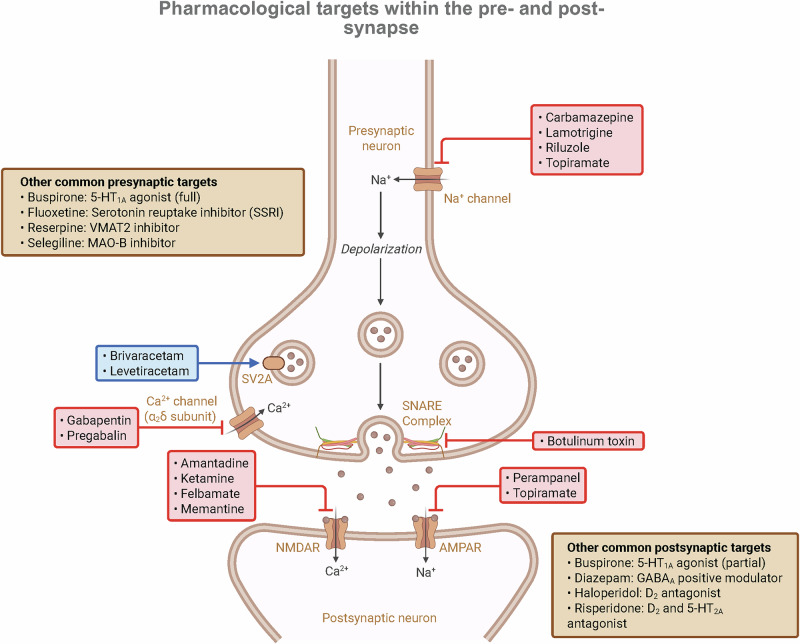
Pharmacological targets within the pre- and post-synapse. Molecular targets of several clinically relevant neuro- and psychotropic drugs. **Presynaptic targets:** (1) Voltage-gated sodium channels: Blockers like carbamazepine, lamotrigine, riluzole, and topiramate reduce neuronal excitability by inhibiting action potential propagation. (2) Voltage-gated calcium channels (VGCCs): Gabapentin and pregabalin bind to the α_2_δ auxiliary subunit of VGCCs. (3) Synaptic vesicle protein 2A (SV2A): Levetiracetam and brivaracetam bind to SV2A, a protein involved in regulating the function and trafficking of the synchronous calcium sensor Syt-1. (4) SNARE complex: Botulinum toxin proteolytically cleaves SNARE proteins (e.g., SNAP-25, VAMP2), directly preventing vesicle fusion. Other common presynaptic targets include the serotonin reuptake transporter (SERT), monoamine oxidase (MAO), and the vesicular monoamine transporter 2 (VMAT2). **Postsynaptic targets:** (5) NMDA receptors (NMDARs): Antagonists like amantadine, ketamine, felbamate, and memantine block NMDAR-mediated currents. (6) AMPA receptors (AMPARs): Antagonists like perampanel and topiramate block AMPAR-mediated currents. Other common postsynaptic targets include the 5-HT_1A_ receptor, GABA_A_ receptors, and dopamine (D_2_) and serotonin (5-HT_2A_) receptors.

Further highlighting the importance of the presynaptic terminal in epilepsy is the synaptic vesicle protein SV2A, the molecular target of the widely used anti-seizure medication levetiracetam [250]. While most anti-seizure medications act by modulating ion channels to reduce excitability, levetiracetam represents a mechanistically distinct approach by targeting the presynaptic release machinery (Fig. 4). SV2A is critically involved in regulating the function and trafficking of Syt-1 [251]. Human mutations in SV2A that cause intractable epilepsy have been shown to disrupt this interaction and lead to mislocalization of Syt-1 and impaired control of synchronous release [251]. This provides a direct link between a key therapeutic target, the core release machinery, and the pathophysiology of epilepsy.

### Schizophrenia and synaptic dysfunction

The “synaptic hypothesis” of schizophrenia asserts that the disorder arises from disrupted synaptic connectivity and information processing [252–254]. A substantial body of evidence points to presynaptic pathology as a key contributor. Post-mortem studies of brains from individuals with schizophrenia have consistently revealed reductions in the levels of key presynaptic proteins in critical brain regions like the dorsolateral prefrontal cortex and hippocampus [255]. The most consistently observed finding is a reduction in synaptophysin, a general marker of synaptic density [255]. Beyond this general marker, specific deficits have been found in proteins that directly regulate vesicle pool and release probability. For example, multiple studies have reported decreased expression of Syn II [256, 257]. Genetic studies have also implicated the *Syn II* gene as a risk factor for schizophrenia [258]. These molecular deficits in the presynaptic machinery are thought to underlie the irregular neural circuit activity and cognitive impairments that characterize the disorder [259]. Current treatments for schizophrenia, such as antipsychotic medications, aim to block or modulate the activity of neurotransmitter receptors to correct these disruptions and alleviate symptoms (Fig. 4) [260, 261].

### Parkinson’s disease and α-synuclein

In Parkinson’s disease, the pathological aggregation of the presynaptic protein α-synuclein into Lewy bodies is a defining hallmark [262]. However, before it aggregates, α-synuclein plays a complex role in regulating neurotransmitter release. Under physiological conditions, it is thought to function as a SNARE complex chaperone by binding to VAMP2 and promoting the assembly of the fusion machinery [263]. In contrast, the overexpression and oligomerization of α-synuclein, which occurs in Parkinson’s disease, has an inhibitory effect on synaptic transmission [264]. This pathological gain-of-function can impair neurotransmitter release through several proposed mechanisms. Some studies suggest that oligomeric α-synuclein directly interferes with SNARE-complex assembly and vesicle docking [265–267], while others propose an indirect mechanism whereby α-synuclein sequesters lipid modulators, such as arachidonic acid, that are required for efficient SNARE-mediated fusion [268]. This dual role of α-synuclein places it at a critical intersection between normal synaptic function and neurodegeneration. Treatment strategies for Parkinson’s disease often aim to restore dopaminergic activity, primarily using the dopamine precursor levodopa, dopamine agonists, or monoamine oxidase B (MAO-B) inhibitors, which all help manage motor symptoms by compensating for the impaired synaptic release caused by α-synuclein aggregation (Fig. 4) [269–271].

## Future directions and therapeutic implications

The elucidation of distinct modes of neurotransmitter release, namely synchronous, asynchronous, spontaneous, and neuromodulatory, has profoundly advanced our understanding of neuronal communication. Furthermore, recognizing that dysfunction in the molecular machinery governing these modes is central to the pathophysiology of numerous brain disorders provides a clear roadmap for developing novel, mechanism-based therapies. However, several questions remain. The potential to selectively target these diverse release mechanisms in neurological and psychiatric disorders characterized by aberrant synaptic signaling opens exciting new avenues for therapeutic intervention. Many current pharmacological treatments for these diseases largely target neurotransmitter receptors or transporters (Fig. 4), often leading to broad, system-wide effects and significant side-effect profiles. Targeting the presynaptic release machinery specific to a particular mode of release could offer greater precision and efficacy. For example:

- Selective modulators of release machinery: A key future direction involves developing drugs that can selectively enhance or inhibit modes of release by targeting their unique molecular components. For example, in disorders of network hyperexcitability such as epilepsy, which may involve an imbalance where excitatory asynchronous release is enhanced or tonic inhibitory asynchronous release is impaired, compounds that selectively dampen the function of calcium sensors like Syt-7 or Doc2α, or enhance the clamping function of Syt-1, could provide novel therapeutic benefits.
- Targeting spontaneous release for mood disorders: The rapidly acting antidepressant ketamine, which in part acts by inhibiting spontaneous NMDAR activation, highlights the immense therapeutic potential of targeting this release mode. Identifying other pathways that specifically regulate spontaneous release or its downstream signaling could yield a new generation of rapid-acting antidepressants.
- Fine-tuning neuromodulatory systems: For neuromodulators like dopamine and serotonin, future therapies could aim to restore a healthy balance between tonic and phasic signaling, rather than simply increasing or decreasing overall transmitter levels. Such interventions would provide greater temporal and spatial specificity to potentially reduce the off-target effects common to traditional pharmacological strategies.

In conclusion, the journey from viewing synaptic transmission as a singular, AP-driven event to recognizing a rich tapestry of dynamic release modes has been transformative for neuroscience. Each mode, with its unique molecular signature and temporal dynamics, contributes in distinct ways to the intricate processes of neuronal signaling, plasticity, and computation. The elucidation of release mode specific mechanisms and function is not only fundamental to our understanding of brain function but also critical for developing the next generation of targeted and effective therapies for a wide range of debilitating disorders. By moving beyond a simple receptor-centric view to embrace the complexity of presynaptic release machineries, molecular psychiatry and neuroscience are poised to make significant advances in the years and decades to come.
